# Pediatric Lupus Presenting as Pulmonary Hypertension, Myocarditis, and Massive Pericardial Effusion in an 11-Year-Old Girl: A Case Report and Literature Review

**DOI:** 10.3389/fped.2022.772422

**Published:** 2022-01-26

**Authors:** Yu-Jhen Chen, Ying-Jui Lin, Mindy Ming-Huey Guo

**Affiliations:** ^1^Department of Pediatric Allergy Immunology and Rheumatology, Kaohsiung Chang Gung Memorial Hospital and Chang Gung University College of Medicine, Kaohsiung, Taiwan; ^2^Department of Pediatric Cardiology, Kaohsiung Chang Gung Memorial Hospital and Chang Gung University College of Medicine, Kaohsiung, Taiwan

**Keywords:** systemic lupus erythematosus (SLE), myocarditis, pulmonary arterial hypertension (PAH), children, pericardial effusion (PE)

## Abstract

Systemic lupus erythematosus (SLE) is an autoimmune disease that may cause vital organ damage. Although not rare for child-onset SLE to have cardiovascular or pulmonary involvement, myocarditis, and pulmonary hypertension are infrequent features and can be life-threatening. In this case report, we describe an 11-year-old girl with SLE who initially presented with fulminant myocarditis pulmonary hypertension, and massive pericardial effusion. Initial immunosuppressive therapy with methylprednisolone pulse therapy, and IVIG were administered, followed by cyclophosphamide, which was ultimately successful, with no residual pulmonary hypertension and no recurrence of myocarditis for over 3 years after the initial episode. Our case highlights the need for clinicians to be aware of systemic lupus erythematosus as a possible diagnostic entity in pediatric patients with severe myocarditis or pulmonary hypertension. Aggressive immunosuppressive therapy should be strongly considered in such cases, as it may lead to good short-term and long-term outcomes.

## Introduction

**Figure 1:**
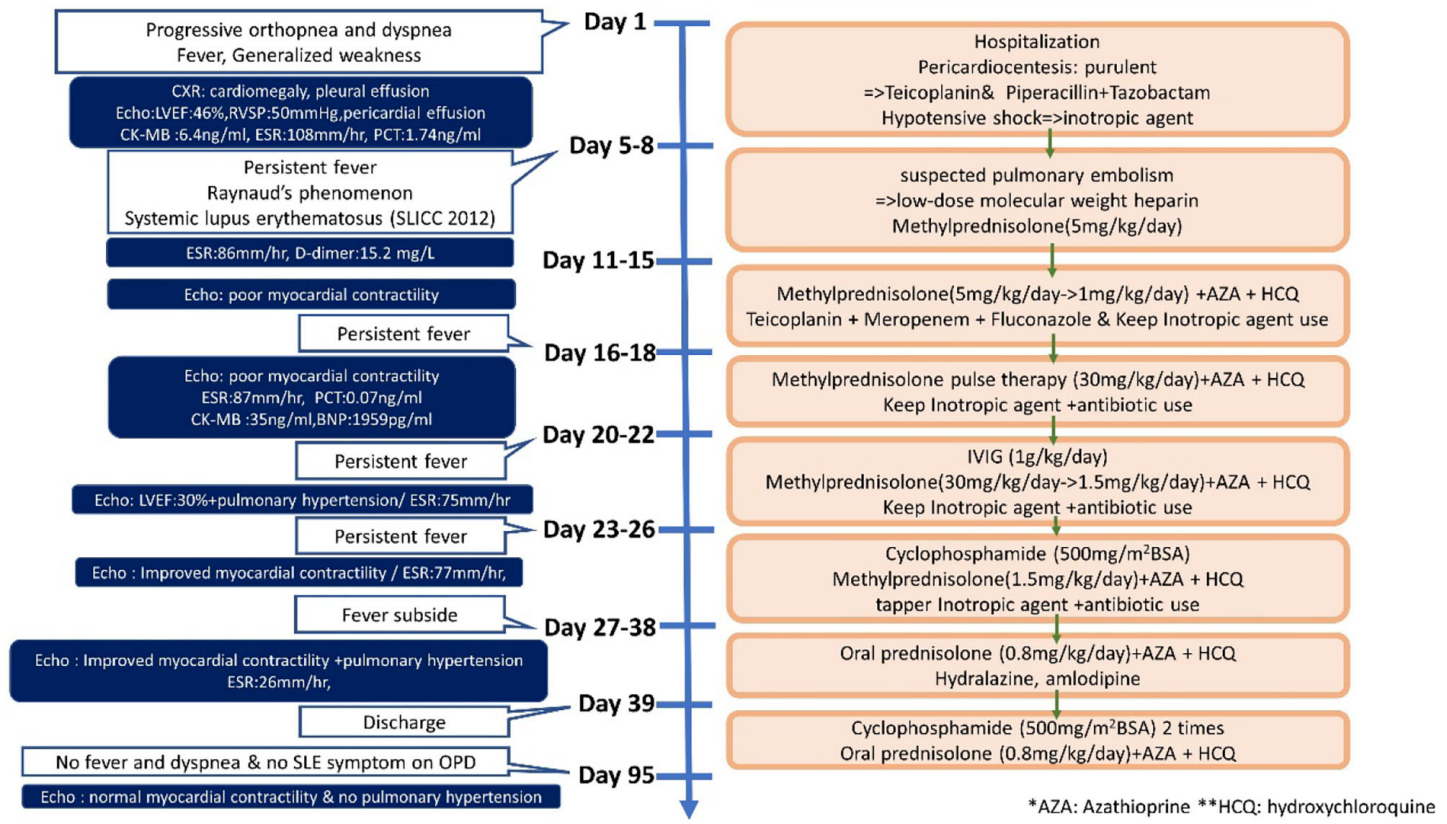
Timeline of this case report.

Systemic lupus erythematosus (SLE) is an autoimmune disease that involves multiple organs *via* immune processes (1). The prevalence of pediatric lupus ranges from 3.3 to 24 per 100,000 children. The disease course of patients with lupus diagnosed in childhood tends to be more severe and more likely to damage the musculoskeletal, ocular, renal, and neuropsychiatric systems when compared to patients with the adult-onset disease (2, 3). Cardiac involvement is frequently seen in SLE, with pericarditis being the most common manifestation. Myocarditis, valve abnormalities, and coronary artery disease may also develop in SLE (4). Moreover, SLE affects the pulmonary system, causing pleural, parenchymal, and pulmonary vascular damage, and it is the second most common cause of connective-tissue disease related pulmonary hypertension, after scleroderma (5). Although not rare for child-onset SLE to have cardiovascular or pulmonary involvement, myocarditis, and pulmonary hypertension are infrequent and can be life-threatening (6). In this case report, we describe a child who first presented with myocarditis with massive pericardia effusion and pulmonary hypertension at the time of pediatric SLE diagnosis (Figure 1).

## Case presentation

**Figure 2:**
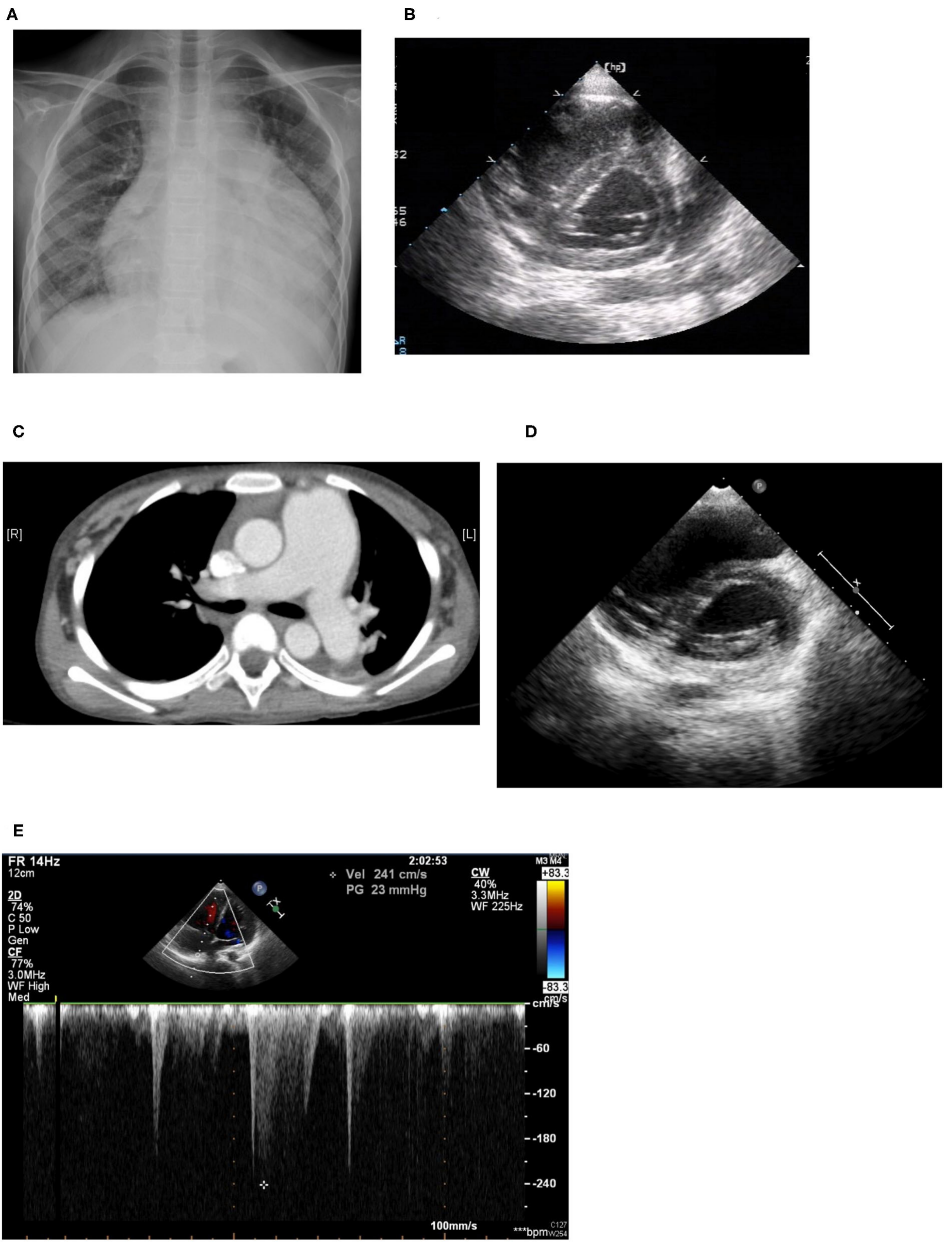
**(A)** Cardiomegaly and pleural effusion seen on chest x-ray on admission. **(B)** Echocardiogram on admission: Right ventricle dilatation due to pulmonary hypertension with shift of the intraventricular septum to the left, resulting in a D-shaped left ventricle (D-sign). **(C)** Chest CT: Dilated pulmonary arteries. **(D)** Echocardiogram performed 1 year later: improving pulmonary hypertension with resolution of “D-sign.” **(E)** Echocardiogram performed 1 year later: Improving pulmonary hypertension with tricuspid regurgitation with maximum velocity of 2.41 m/s, estimated right ventricle systolic pressure 30 mmHg.

An 11-year-old girl initially came to our hospital with shortness of breath, worsening malaise, and general weakness for 2 days. She had been taken to a clinic 2 days prior to admission for an episode of syncope that resolved after 2 min. However, due to progressive orthopnea and dyspnea on exertion, she was brought to our emergency department for additional evaluation. Upon further inquiry, the patient mentioned having intermittent migratory arthralgia of the bilateral ankles, knees and cervical joints for the past 8 months. Over the last 6 months, parents also noted that the patient had occasional episodes of tactile fever about once a month, general malaise and body weight loss of 2 kg. On physical examination at the emergency department the patient was found to be weak but responsive, with severe cachexia, a height of 140 cm, and a weight of only 23 kg. Vital signs on arrival showed hypotension (86/49 mmHg), tachycardia (163 beats/min), tachypnea (respiratory rate 29 breaths/min), temperature of 38.8°C with an oxygen saturation of 98% on room air. She was also found to have suprasternal retractions, rales in the bilateral lung fields and cold and clammy extremities. Initial laboratory values showed microcytic anemia (hemoglobulin: 11.3 g/dL, MCV: 74.9 fL), elevated liver enzymes (AST/ALT:103/53 U/L), hypoalbuminemia (2.73 g/dL), elevated CK-MB (6.4 ng/mL), and procalcitonin (1.74 ng/mL). Troponin I and renal function (Creatinine: 0.42 mg/dl) were within normal range. Neither hyperglycemia nor hyperthyroidism was noted in laboratory values. Cardiomegaly and bilateral pleural effusion were observed on a chest X-ray. Echocardiography showed impaired left ventricle (LV) performance [left ventricular ejection fraction (LVEF) was 46%], massive pericardial effusion, and pulmonary hypertension (estimated right ventricular systolic pressure around 50 mmHg; Figures 2A,B).

The patient was then transferred to the pediatric intensive care unit where echo-guided pericardiocentesis of the massive pericardial effusion was performed twice in the initial 2 days. A total of 548 ml of turbid greenish pericardial fluid was drained, and further analysis showed that the fluid was highly purulent with a white blood cell count of 128,700 cells/μL, 85% neutrophils, a specific gravity of 1.0038, protein of 7.0 g/dL, LDH of 3,860 U/L, and glucose of 3 mg/dL, but gram stain, acid-fast stain, and aerobic and anaerobic cultures of the pericardial fluid were all found to be negative. Due to the highly purulent nature of the pericardial fluid, the patient was initially started on teicoplanin and piperacillin/tazobactam for probable infective pericarditis which was continued for a total of 1 month. Furthermore, the patient was given a colloid infusion with 10% albumin and was started on inotropic agents including dopamine and milrinone for hypotensive shock.

Due to the patient's history of arthralgia, fever, and serositis (including pericarditis and pleural effusion), although she had no family history of autoimmune disease, the patient was further evaluated for the presence of any underlying rheumatic disease. Antinuclear antibodies (ANA) were positive (>1:1,280) and anti-dsDNA titers were 399.7 IU/ml (normal <92.6 IU/ml); the patient also had hypocomplementemia (C3 45.2 mg/dL, C4 13.2 mg/dL), and we found that both anti-RNP and anti-Smith antibodies were positive. The patient was diagnosed with systemic lupus erythematosus (SLE) according to the Systemic Lupus International Collaborating Clinics (SLICC) 2012 criteria for SLE (7). Thereafter, the patient was started on intravenous methylprednisolone for suspected lupus flare.

Sudden onset of tachypnea and tachycardia occurred on the fourth day of admission. Bedside echocardiography was performed again and showed pulmonary hypertension with engorgement of the right atrium and the pulmonary artery and an ejection fraction of 30%. Raynaud's phenomenon with coldness and pallor of the fingers and toes was also noted that day, with swelling in the fifth right toe, which soon became cyanotic, although pulse of the right dorsalis pedis artery was still palpable. We checked D-dimer and found it to be high, with a value of 15.20 mg/L with normal fibrinogen levels and no prolongation of prothrombin time or activated partial thromboplastin time. Lupus anticoagulant was slightly prolonged at 47.6 s (normal range 31–44 s), although anti-β2 glycoprotein antibodies, and anti-cardiolipin antibodies were not detected. The patient was then started on low-dose molecular weight heparin for suspected pulmonary embolism. However, low-dose molecular weight heparin was discontinued after performing a CT-angiogram that showed dilated pulmonary arteries (main pulmonary artery diameter 37 mm, right pulmonary artery 19 mm, left pulmonary artery 18 mm), and dilated right atrium and ventricle but no signs of thrombus within the pulmonary arteries (Figure 2C).

The patient still remained febrile after 2 weeks of antibiotics and pericardial drainage. Therefore, we checked lab values again, which showed that while procalcitonin levels had normalized (0.07 ng/ml) suggesting that the patient's bacterial pericarditis had improved, signs of myocardial inflammation were still present as evidenced by an elevated CK-MB level of 35 ng/mL, Troponin-I of 0.051 ng/mL, and BNP of 1,959 pg/mL, as well as persistent poor myocardial contractility observed on bedside echo, which required inotropic agents. Methylprednisolone pulse therapy at 1 g per day was then given for 3 consecutive days for suspected lupus-induced myocarditis and pulmonary hypertension. However, the patient's fever and hypotension persisted, so the patient was then given intravenous immunoglobulin (IVIG) at 1 g/kg/day for a total of 2 days. Serial bedside echo was performed again and showed that the patient's pulmonary hypertension and myocardial contractility had improved slightly, but the patient was still hypotensive and remained febrile 1 week after IVIG treatment. Finally, the patient was given one dose of cyclophosphamide 500 mg per body surface area (BSA). Thereafter, the patient's fever declined, her hypotension improved, and the inotropic agents were tapered and then discontinued 1 week later. The patient was then discharged with oral steroids, hydroxychloroquine, hydralazine. and amlodipine. Afterwards, the patient was given an additional two more doses of cyclophosphamide (500 mg/BSA/dose) once a month. Cardiac echocardiography performed after both hydralazine and amlodipine had been discontinued after 1 year showed resolution of pulmonary hypertension (tricuspid regurgitation with maximum velocity of 2.41 m/s, estimated right ventricle systolic pressure 30 mmHg), normalization of right atrium and ventricular size, and good left ventricle ejection fraction (Figures 2D,E). The patient has received regular follow-up at our pediatric clinic for the past 3 years. She has shown some clinical symptoms of lupus, such as intermittent malar rash and proteinuria, during the follow-up period in the outpatient department, but no recurrence of pulmonary hypertension or lupus myocarditis.

## Discussion

Cardiac manifestations of SLE are estimated to be present on echocardiogram in up to 50% of adult patients, although these findings are mostly asymptomatic. Common findings among adult patients include pericarditis, myocarditis, non-bacterial endocarditis, and valvular insufficiency (4, 8). Less is known about the incidence of cardiac manifestations of lupus in the pediatric population, although various small population single-center studies have suggested that the rate of pericarditis may range from 12 to 43% (9, 10). A study of 297 children with pediatric lupus within the United States, found that the most common cardiac manifestation was pericarditis (10.4%), followed by valvular insufficiency (9.1%), and less commonly myocarditis (1.0%) and endocarditis (1.0%). Furthermore, cardiac manifestations were most likely to occur within the first year after pediatric lupus was diagnosed, and was associated with African American race and nephritis. The authors also found that cardiac involvement in child-onset SLE, including pericarditis and myocarditis, had a 4.4-fold higher incidence rate than adult-onset SLE and required more aggressive management, such as pericardial drainage (11).

Diagnosing lupus myocarditis primarily relies on clinical features. Patients with SLE-related myocarditis may present with fever, chest pain, tachypnea, tachycardia, gallop rhythm and other arrhythmias, or heart failure. Common echocardiographic findings include unexplained ventricular dysfunction, cardiac chamber enlargement, and hypokinesia of the myocardium (12). Elevation of cardiac enzymes like CK-MB and Troponin I are present in roughly 75% of patients (13). Pathology results from endomyocardial biopsy may help clinicians to confirm the diagnosis, but are not commonly applied in clinical settings and have a low diagnostic yield of around 10–30% (14). However, in this case report, discriminate between infectious pericarditis and lupus myocarditis is difficult at first. Lupus myocarditis and infectious pericarditis have several common features, including the presence of pericardial effusion and decreased myocardial contractility. In fact, purulent pericarditis is a rare complication in SLE but can cause serious illness that progresses rapidly to cardiac tamponade and death (15, 16). Since this patient initially presented with fever, massive purulent pericardial effusion, and mildly elevated procalcitonin levels, we initially treated the patient with empirical antibiotics. After such antibiotics therapy, the patient's pericardial effusion was resolved, and procalcitonin levels normalized, although cultures of the pericardial fluid were negative, suggesting the presence of infection. Nevertheless, the patient remained febrile with poor myocardial contractility and elevated cardiac enzymes even after procalcitonin levels had normalized after 2 weeks of antibiotic therapy. Several immunosuppressive therapies for suspected lupus myocarditis were then administered to the patient, including methylprednisolone, IVIG, and cyclophosphamide, after which the patient's myocardial function improved, and inotropic agents were no longer required. Due to the patient's response to both antibiotics and subsequent immunosuppressive therapy, we believe that lupus myocarditis complicated with massive pericardial effusion and secondary infectious pericarditis is the most likely diagnosis in our patient.

It is also noteworthy that the patient experienced impressive weight loss in this episode. She had body weight loss of 2 kg in the previous 6 months and weighed only 23 kg with mild muscle atrophy on initial presentation. Cachexia is clinically defined by an involuntary edema-free weight loss of at least 5% within a period of 12 months or less in patients with chronic diseases (e.g., heart failure, cancer, COPD, and chronic kidney disease) (17). In addition to heart failure, other factors that influence the patient's body composition, such as metabolic dysfunction, neurologic disorder, malignancy, gastrointestinal problem, or reduced food intake, should be excluded. Initially, we surveyed thyroid function and blood sugar to rule out such metabolic problems as hyperthyroidism and diabetes mellitus, and lab data revealed normal liver and renal function. Neurologic and psychosocial disorders were less likely after we took the patient's history and completed the physical examination. Image findings showed no obvious malignancy lesion. The patient regained her weight in 1 week after starting treatment for lupus myocarditis and had appropriately gained weight during the 2-year follow-up in the outpatient department. Therefore, heart failure due to lupus myocarditis seems to be the mostly likely cause of cachexia in this patient.

Treatment of SLE myocarditis depends on empirical experience and supportive care. In a review of published observational cohorts of 147 adult patients with lupus myocarditis, corticosteroids were used in almost all patients (95.7%) at a dose of 0.5–1 mg/kg/day. Other therapeutic regimens most commonly included intravenous methylprednisolone pulse therapy (500–100 mg/day for 3 days) in 65.8% of patients (13); other immunosuppressants frequently prescribed include cyclophosphamide, azathioprine, mycophenolate mofetil, IVIG, and rituximab (12, 18–20). However, reports regarding therapeutic treatment of lupus myocarditis in children have been limited. We searched PubMed for articles that described the treatment of childhood-onset lupus myocarditis and summarized them in Table 1 (21–24). In total five pediatric patients with lupus myocarditis ranging from 11 to 18 years of age were found in our literature review. In all five, the patients were initially given methylprednisolone pulse therapy; three patients received additional cyclophosphamide, and one patient received rituximab, and another received IVIG. One of the patients died 8 days after admission from multiorgan dysfunction and cardiogenic shock.

**Table 1:** Previous case reports of myocarditis in patients with pediatric lupus.

**Author**	**Age (years)**	**Gender**	**Disease duration**	**Presentation**	**Echocardiography findings**	**Treatment**	**Outcome**
Aggarwal et al. (21)	12	F	NA	Polyarticular arthritis, fever, edema, and painless oral ulcers	Dilated RV and LV, decreased myocardial contractility with LVEF only 30%	· MP pulse (30 mg/kg/dose) daily	LVEF improved; asymptomatic and on tapering doses of oral prednisolone along with hydroxychloroquine
						· Cyclophosphamide pulses (750 mg/m^2^/dose) monthly for 6 months, then every 3 months	
						· Rituximab (375 mg/m^2^/dose) weekly for 4 weeks	
Gupta et al. (22)	12	F	2 years	Fever, malar rash, photosensitivity, tachycardia, pallor, alopecia, and oral ulcers	Moderate mitral regurgitation, mild tricuspid regurgitation, and global hypokinesia with a LVEF of 56%	· MP pulse followed by oral prednisolone at a dose of 2 mg/kg/day	Asymptomatic with an LVEF of 72% on echocardiography
	11	F	6 months	Pain in the small joints, low grade fever, cough for 1 month, generalized edema, malar rash, oral ulcers, and tachycardia	Moderate mitral regurgitation, trivial tricuspid regurgitation, and global hypokinesia with an LVEF of 18%	· MP pulse and cyclophosphamide pulse	Expired after 8 days in hospital (multiorgan dysfunction due to shock)
Huang et al. (23)	12	F	At presentation	Sudden onset chest pain, tachycardia, tachypnea, and cyanosis	Enlargement of all four chambers with decreased LVEF 43.4%, pericardial effusion and severe mitral regurgitation	· MP pulse 1 g/day for 3 days and then prednisolone 2 mg/kg/day equivalent dose)	Improved LVEF 55% and resolution of chamber enlargement except left ventricular enlargement only
						· Azathioprine and hydroxychloroquine	
Suri et al. (24)	18	M	6 years	Right femoropopliteal deep vein thrombosis and pallor	Global LV hypokinesia with LVEF of 43%	· MP pulses (1 g/day) for 3 days followed by oral prednisolone 60 mg/day	Tachycardia, dyspnea, and crepitations decreased
						· Cyclophosphamide pulse 1 g for 1 dose)	LVEF of 50%
						· IVIG (400 mg/kg/day) for 5 days	

*RV, right ventricle; LV, left ventricle; LVEF, left ventricular ejection fraction; MP, methylprednisolone; IVIG, intravenous immunoglobulin*.

Pulmonary artery hypertension is a relatively rare complication of lupus, with an estimated incidence rate of <1% in the UK and about 2% in Taiwan (25); it is a relatively poor prognostic finding with a 5-year survival rate of only 68% (26). Pulmonary arterial hypertension (PAH), is defined as mean pulmonary arterial pressure (mPAP) ≧ 25 mmHg. Common symptoms include dyspnea on exertion, chest pain, syncope, and other signs of right heart failure. Pulmonary hypertension in the lupus setting is associated with positive anti-ribonucleoprotein antibody titers, anti-phospholipid syndrome and Raynaud's phenomenon (26).

Therapeutic treatment of PAH in the lupus setting can be split into two categories: vasodilators or immunosuppressants. Of the common immunosuppressants used in lupus, cyclophosphamide has been the most widely studied and has found to be efficacious in several small-scale observational studies in adults. Common dosage regimens include cyclophosphamide (500–1,000 mg/m^2^/month, for 3–10 months) usually in combination with oral prednisolone (0.5–1 mg/kg/day for 4 weeks with tapering) (27). Alternative treatment options include rituximab in patients with refractory pulmonary hypertension (28), or mycophenolate mofetil and cyclosporine (29). Pulmonary vasodilator therapy usually starts with such calcium channel blockers as nifedipine, amlodipine etc. as first-line agents (30). Vasodilator therapy has also been found to be effective in several double blind, placebo-controlled studies in adults. Bosentan, an endothelin receptor antagonist, sildenafil, a phosphodiesterase type 5 inhibitor, and treprostinil, a vasodilator, have all been found to improve exercise capacity, hemodynamics, and heart function class in double-blind placebo controlled trials of patients with connective tissue disease and pulmonary arterial hypertension, which included a small subset of patients diagnosed with SLE (31–33). Vasodilator add-on therapy has also been shown to be effective in patients with severe lupus-associated pulmonary hypertension who failed to respond to immunosuppressants alone (34).

Treatment of PAH in the pediatric lupus setting has been mostly empirical and extrapolated from studies performed in adults. After a literature review, we identified two case reports that mentioned the treatment of pediatric SLE with PAH which are listed in Table 2 (35, 36). One was a 7-year-old female who showed good response to cyclophosphamide pulse therapy monthly for the first 6 months, which was then followed by cyclophosphamide pulse therapy every 3 months in conjunction with steroids and mycophenolate mofetil (35). In comparison, a 10-year-old female who received steroids and low-dose aspirin alone for 3 weeks and showed no signs of improvement (36).

**Table 2:** Previous case reports of pulmonary hypertension in patients with pediatric lupus.

**Author**	**Age (years)**	**Gender**	**Disease duration**	**Presentation**	**Image findings**	**Treatment**	**Outcome**
Beresford et al. (35)	7	F	NA	NA	· Chest CT: interstitial lung disease	· Cyclophosphamide (500~1,000 mg/m^2^ monthly for 6 months, and then 3 monthly, as clinically indicated;) and Azathioprine (2 mg/kg daily), but poor response	Maintained on MMF and high dose corticosteroids with significant steroid toxicity
					· Lung biopsy: chronic inflammatory changes and follicular bronchiolitis	· MP pulse (30 mg/kg/dose daily for 3 days, followed by weekly pulses, or as clinically indicated) and MMF (600 mg/m^2^ twice daily to a maximum of 2 g)	
					· Echocardiography: significant pulmonary hypertension		
Khetarpal et al. (36)	10	F	At presentation	Progressive breathlessness present for 8 months, fatigue, generalized edema, and malar rash photosensitivity	Cardiac catheterization: severe pulmonary arterial hypertension (left pulmonary artery pressure of 90/44/160 mm Hg and main pulmonary artery pressure 95/45/62 mm Hg)	prednisolone (2 mg/kg) and low dose aspirin (5 mg/kg)	Not show any improvement after 3 weeks of therapy and did not come for follow up after discharge

*MP, methylprednisolone; MMF, mycophenolate mofetil*.

In this case report, we describe an 11-year-old female with freshly diagnosed lupus who presented with pulmonary hypertension and myocarditis and massive pericardial effusion. Initially, immunosuppressive therapy with methylprednisolone pulse therapy and then IVIG were administered, and finally cyclophosphamide was ultimately successful, with no residual pulmonary hypertension and no recurrence of myocarditis for over 3 years after the initial episode. Our case highlights the need for clinicians to be aware of systemic lupus erythematosus as a possible diagnostic entity in pediatric patients with severe myocarditis or pulmonary hypertension. Aggressive immunosuppressive therapy should be strongly considered in such cases, and may lead to both good short-term and good long-term outcomes.

## Data Availability Statement

The original contributions presented in the study are included in the article/supplementary material, further inquiries can be directed to the corresponding author/s.

## Author Contributions

MG and Y-JL contributed to the conception and design of the study. MG and Y-JC organized the database. Y-JC wrote the first draft of the manuscript. MG wrote sections of the manuscript. All authors contributed to manuscript revision and read and approved the submitted version.

## Conflict of Interest

The authors declare that the research was conducted in the absence of any commercial or financial relationships that could be construed as a potential conflict of interest.

## Publisher's Note

All claims expressed in this article are solely those of the authors and do not necessarily represent those of their affiliated organizations, or those of the publisher, the editors and the reviewers. Any product that may be evaluated in this article, or claim that may be made by its manufacturer, is not guaranteed or endorsed by the publisher.
